# Supplementary material for: Application of a 1H brain MRS benchmark dataset to deep learning for out-of-voxel artifacts
Source: Imaging Neurosci (Camb). 2023 Nov 2;1:imag-1-00025. doi: 10.1162/imag_a_00025 (PMC12007519; doi:10.1162/imag_a_00025)
Supplement: Supplementary Material [file imag_a_00025-supp.pdf]

Supplemental Material:

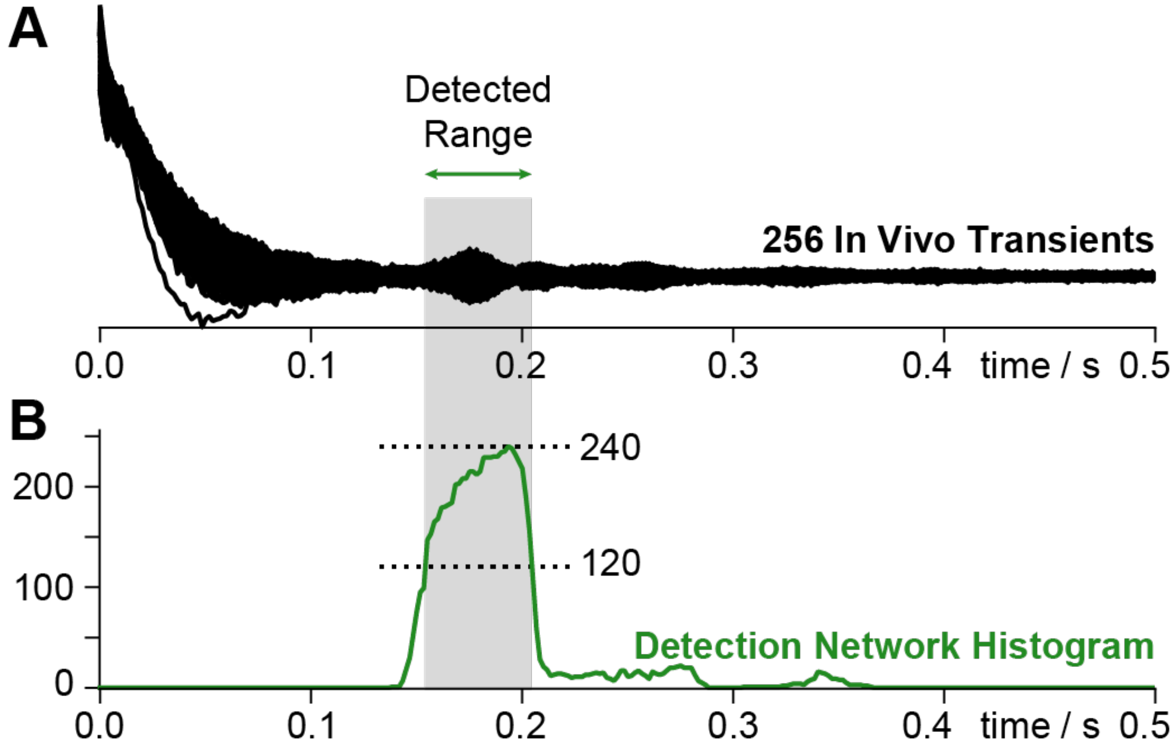

Supplemental Figure 1: Time-domain window (gray) used to calculate the fractional reduction in standard deviation for the *in vivo* transients. **A)** Each of the 256 MEGA-PRESS transients (128 Edit-on and 128 Edit-Off) overlaid. **B)** Histogram (green) showing the total number of detections by the Detection Network across each timepoint. This window was established algorithmically by using 50% of the maximum count as a threshold for the window.

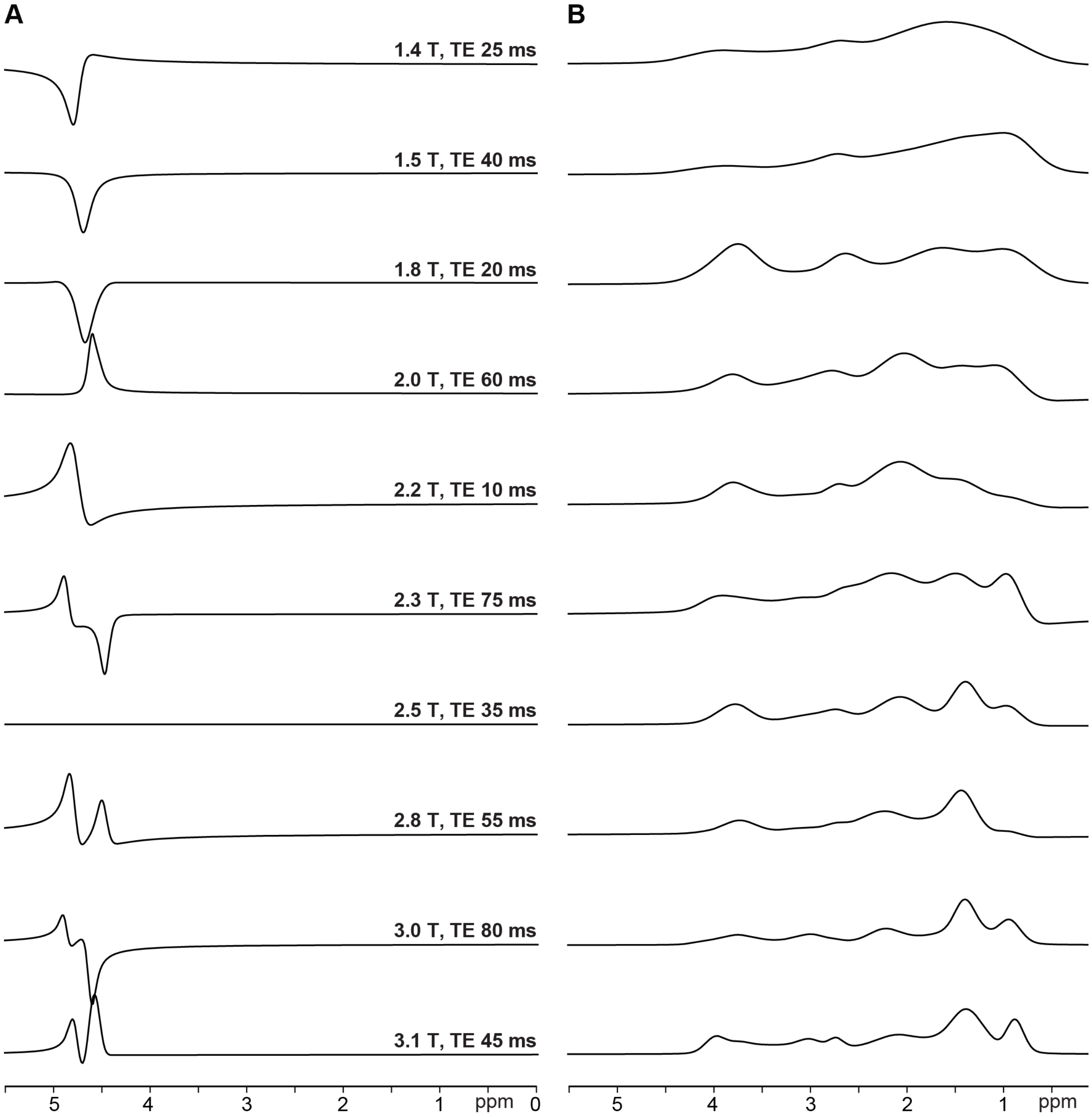

Supplemental Figure 2: Ten representative examples of **A)** residual water and **B)** macromolecule components. Examples match the full spectra shown in Figure 3; each spectrum is scaled independently for visualization.

B_0 (T)	Every Point	Every 2nd Point	Every 3rd Point	Every 4th Point	Every 5th Point	Every 6th Point	Every 7th Point	Every 8th Point
1.4	3733.33	1866.67	1244.44	933.33	746.67	622.22	533.33	466.67
1.5	4000.00	2000.00	1333.33	1000.00	800.00	666.67	571.43	500.00
1.6	4266.67	2133.33	1422.22	1066.67	853.33	711.11	609.52	533.33
1.7	4533.33	2266.67	1511.11	1133.33	906.67	755.56	647.62	566.67
1.8	4800.00	2400.00	1600.00	1200.00	960.00	800.00	685.71	600.00
1.9	5066.67	2533.33	1688.89	1266.67	1013.33	844.44	723.81	633.33
2.0	5333.33	2666.67	1777.78	1333.33	1066.67	888.89	761.90	666.67
2.1	5600.00	2800.00	1866.67	1400.00	1120.00	933.33	800.00	700.00
2.2	5866.67	2933.33	1955.56	1466.67	1173.33	977.78	838.10	733.33
2.3	6133.33	3066.67	2044.44	1533.33	1226.67	1022.22	876.19	766.67
2.4	6400.00	3200.00	2133.33	1600.00	1280.00	1066.67	914.29	800.00
2.5	6666.67	3333.33	2222.22	1666.67	1333.33	1111.11	952.38	833.33
2.6	6933.33	3466.67	2311.11	1733.33	1386.67	1155.56	990.48	866.67
2.7	7200.00	3600.00	2400.00	1800.00	1440.00	1200.00	1028.57	900.00
2.8	7466.67	3733.33	2488.89	1866.67	1493.33	1244.44	1066.67	933.33
2.9	7733.33	3866.67	2577.78	1933.33	1546.67	1288.89	1104.76	966.67
3.0	8000.00	4000.00	2666.67	2000.00	1600.00	1333.33	1142.86	1000.00
3.1	8266.67	4133.33	2755.56	2066.67	1653.33	1377.78	1180.95	1033.33

Supplemental Table 1: Field strengths (*Tesla*) and possible spectral widths (*Hertz*) available using the AGNOSTIC basis sets. These combinations are achievable by subsampling the time-domain from every timepoint to every 8th timepoint and allows for maintaining a minimum of 2048 timepoints. Each of these combinations is available for the 15 echo times, from 10 ms to 80 ms, in steps of 5 ms.

	Synthetic Range (mM)	
**Metabolite**	**Low**	**High**
Acetate	0.00	0.00
Alanine*	0.47	0.77
Ascorbate	0.36	1.53
Aspartate	0.00	4.66
Adenosine Triphosphate	0.00	0.00
β-Hydroxybutyrate	0.00	0.00
β-Hydroxyglutarate	0.00	0.00
Citrate	0.00	0.00
Creatine	1.41	10.50
Cysteine	0.00	0.00
Ethanol Amine	0.00	0.00
Ethyl Alcohol	0.00	0.00
γ-Amino Butyric Acid	0.52	1.99
Glucose*	0.94	1.53
Glutamine	0.26	3.64
Glutamate	3.88	13.17
Glycerophosphocholine	0.05	5.00
Glutathione	0.16	2.41
Glycine*	0.94	1.53
Glycerol	0.00	0.00
Histamine	0.00	0.00
Histidine	0.00	0.00
Homocarnosine	0.00	0.00
Lactate	0.00	1.44
Macromolecule 0.92	1.00	30.00
Macromolecule 1.21	1.00	8.00
Macromolecule 1.39	1.00	35.00
Macromolecule 1.67	1.00	15.00
Macromolecule 2.04	1.00	35.00
Macromolecule 2.26	1.00	20.00
Macromolecule 2.56	1.00	5.00
Macromolecule 2.70	1.00	7.00
Macromolecule 2.99	1.00	10.00
Macromolecule 3.21	1.00	7.00
Macromolecule 3.62	1.00	5.00
Macromolecule 3.75	1.00	10.00
Macromolecule 3.86	1.00	4.00
Macromolecule 4.03	1.00	7.00
Myo-inositol†	2.08	14.00
N-Acetylaspartate†	5.38	18.00
N-Acetylaspartylglutamate†	0.26	2.26
Phosphocholine*	0.01	2.00
Phosphocreatine*	3.38	6.44
Phosphoethanolamine*	1.41	2.30
Phosphoethyl Alcohol	0.00	0.00
Phenylalanine	0.00	0.00
Scyllo-inositol	0.00	0.39
Serine	0.00	0.00
Taurine	0.00	2.89
Threonine	0.00	0.00
Tryptophan	0.00	0.00
Tyrosine	0.00	0.00
Valine	0.00	0.00

Supplemental Table 2: Concentration ranges for the healthy brain, used to generate synthetic spectra. These mM values were based upon a meta-analysis preliminary to (Gudmundson et al., 2023), with some values (marked *) supplemented from the Fit Challenge ranges (Marjańska et al., 2021) and other ranges (marked †) extended to offer greater flexibility. Concentrations were sampled uniformly between the low and high values to generate the synthetic spectra.

Disease / Metabolite	Synthetic Range Low	High
Seizure		
Creatine	0.918	1.012
Phosphocreatine	0.918	1.012
Glycerophosphocholine	0.731	1.147
Phosphocholine	0.731	1.147
γ-Amino Butyric Acid	0.930	1.173
Glutamate	0.787	1.247
Glutamine	0.787	1.247
Glutathione	0.887	1.243
Myo-inositol	0.802	1.134
N-Acetylaspartate	0.751	1.002
N-Acetylaspartylglutamate	0.751	1.002
Stroke		
Creatine	0.684	1.146
Phosphocreatine	0.684	1.146
Glycerophosphocholine	0.855	1.527
Phosphocholine	0.855	1.527
Glutamate	0.874	1.140
Glutamine	0.874	1.140
Lactate	1.000	6.922
Myo-inositol	0.827	1.265
N-Acetylaspartate	0.727	1.074
N-Acetylaspartylglutamate	0.727	1.074
Traumatic Brain Injury		
Aspartate	0.785	0.910
Creatine	0.814	1.162
Phosphocreatine	0.814	1.162
Glycerophosphocholine	0.930	1.057
Phosphocholine	0.930	1.057
γ-Amino Butyric Acid	0.860	0.984
Glutamate	0.824	1.214
Glutamine	0.824	1.214
Myo-inositol	0.737	1.315
N-Acetylaspartate	0.795	1.011
N-Acetylaspartylglutamate	0.795	1.011
Type-1 Diabetes		
Aspartate	0.895	1.496
Creatine	0.977	1.039
Phosphocreatine	0.977	1.039
Glycerophosphocholine	1.034	1.140
Phosphocholine	1.034	1.140
Glutamate	0.895	1.216
Glutamine	0.956	1.353
Glutathione	0.872	1.435
Myo-inositol	0.893	1.092
N-Acetylaspartate	0.947	1.008
N-Acetylaspartylglutamate	0.947	1.008
Scyllo-inositol	0.501	0.992
Taurine	0.754	1.322

Metabolite	Synthetic Range Low	High
Cancer		
Creatine	0.256	1.340
Phosphocreatine	0.256	1.340
Glycerophosphocholine	1.139	1.949
Phosphocholine	1.139	1.949
Glutamate	0.780	1.320
Glutamine	0.780	1.320
Lactate	1.00	9.99
Myo-inositol	0.829	1.519
N-Acetylaspartate	0.509	0.956
N-Acetylaspartylglutamate	0.509	0.956
Chronic Pain		
Glycerophosphocholine	0.943	1.285
Phosphocholine	0.943	1.285
γ-Amino Butyric Acid	0.896	1.168
Glutamate	0.790	1.121
Glutamine	0.790	1.121
Myo-inositol	0.942	1.049
N-Acetylaspartate	0.775	1.280
N-Acetylaspartylglutamate	0.775	1.280
Migraine		
Aspartate	0.434	1.409
Creatine	0.921	1.011
Phosphocreatine	0.921	1.011
Glycerophosphocholine	0.959	1.137
Phosphocholine	0.959	1.137
Glutamate	0.841	1.119
Glutamine	0.841	1.119
Myo-inositol	0.866	1.032
N-Acetylaspartate	0.755	1.067
N-Acetylaspartylglutamate	0.755	1.067
Fibromyalgia		
Creatine	0.760	1.429
Phosphocreatine	0.760	1.429
Glycerophosphocholine	0.840	1.236
Phosphocholine	0.840	1.236
γ-Amino Butyric Acid	0.724	0.937
Glutamate	1.005	1.104
Glutamine	0.711	1.107
Myo-inositol	0.844	1.232
N-Acetylaspartate	0.847	1.061
N-Acetylaspartylglutamate	0.847	1.061

…table continued on next page

	Synthetic Range	
**Disease / Metabolite**	**Low**	**High**
Post-Traumatic Stress Disorder		
Creatine	0.940	1.235
Phosphocreatine	0.940	1.235
Glycerophosphocholine	0.843	1.284
Phosphocholine	0.843	1.283
γ-Amino Butyric Acid	0.982	1.059
Glutamate	0.892	1.134
Glutamine	0.892	1.134
Myo-inositol	0.939	1.198
N-Acetylaspartate	0.969	1.156
N-Acetylaspartylglutamate	0.969	1.156
Obsessive Compulsive Disorder		
Creatine	0.890	1.320
Phosphocreatine	0.890	1.320
Glycerophosphocholine	0.784	1.223
Phosphocholine	0.784	1.223
Glutamate	0.868	1.243
Glutamine	0.868	1.243
Myo-inositol	0.743	1.437
N-Acetylaspartate	0.846	1.100
N-Acetylaspartylglutamate	0.846	1.100
Depression		
Creatine	0.938	1.021
Phosphocreatine	0.938	1.021
Glycerophosphocholine	0.741	1.158
Phosphocholine	0.741	1.158
γ-Amino Butyric Acid	0.769	1.400
Glutamate	0.872	1.119
Glutamine	0.894	1.177
Glutathione	0.822	1.082
Myo-inositol	0.874	1.239
N-Acetylaspartate	0.864	1.080
N-Acetylaspartylglutamate	0.864	1.080
Addiction		
Creatine	0.775	1.161
Phosphocreatine	0.755	1.161
Glycerophosphocholine	0.788	1.202
Phosphocholine	0.788	1.202
γ-Amino Butyric Acid	0.669	1.289
Glutamate	0.807	1.229
Glutamine	0.807	1.229
Glycine	0.969	1.335
Glutathione	0.935	1.442
Myo-inositol	0.820	1.135
N-Acetylaspartate	0.761	1.195
N-Acetylaspartylglutamate	0.761	1.195

	Synthetic Range	
**Disease / Metabolite**	**Low**	**High**
Schizophrenia		
Creatine	0.948	1.045
Phosphocreatine	0.948	1.045
Glycerophosphocholine	0.946	1.157
Phosphocholine	0.946	1.157
γ-Amino Butyric Acid	0.732	1.261
Glutamate	0.857	1.164
Glutamine	0.857	1.164
Myo-inositol	0.806	1.239
N-Acetylaspartate	0.910	1.103
N-Acetylaspartylglutamate	0.910	1.103
Psychosis		
Creatine	0.983	1.059
Phosphocreatine	0.983	1.059
Glycerophosphocholine	0.892	1.127
Phosphocholine	0.892	1.127
γ-Amino Butyric Acid	0.725	1.176
Glutamate	0.813	1.172
Glutamine	0.813	1.172
Glycine	1.131	1.423
Glutathione	0.917	1.034
Myo-inositol	0.892	1.090
N-Acetylaspartate	0.910	1.048
N-Acetylaspartylglutamate	0.910	1.048
Personality Disorder		
Creatine	0.961	1.110
Phosphocreatine	0.961	1.110
Glycerophosphocholine	0.925	1.007
Phosphocholine	0.925	1.007
Glutamate	0.949	1.207
Glutamine	0.949	1.207
Glutathione	0.917	1.034
Myo-inositol	0.989	1.081
N-Acetylaspartate	0.880	0.997
N-Acetylaspartylglutamate	0.880	0.997
Bipolar Disorder		
Creatine	0.900	1.061
Phosphocreatine	0.900	1.061
Glycerophosphocholine	0.854	1.269
Phosphocholine	0.854	1.269
Glutamate	0.907	1.115
Glutamine	0.907	1.115
Glutathione	0.957	1.150
Myo-inositol	0.812	1.209
N-Acetylaspartate	0.863	1.109
N-Acetylaspartylglutamate	0.863	1.109

…table continued on next page

Disease / Metabolite	Synthetic Range Low	High	Disease / Metabolite	Synthetic Range Low	High
Multiple Sclerosis			Dementia		
Glycerophosphocholine	0.880	1.077	Ascorbate	1.132	1.231
Phosphocholine	0.880	1.077	Aspartate	1.028	1.168
γ-Amino Butyric Acid	0.851	1.017	Creatine	1.010	1.028
Glutamate	0.887	1.030	Phosphocreatine	1.010	1.028
Glutamine	0.887	1.030	Glycerophosphocholine	0.850	1.150
Glutathione	0.844	1.069	Phosphocholine	0.850	1.150
Myo-inositol	0.892	1.078	γ-Amino Butyric Acid	0.513	1.183
N-Acetylaspartate	0.924	1.044	Glutamate	0.771	1.139
N-Acetylaspartylglutamate	0.924	1.044	Glutamine	0.955	1.172
Parkinson's Disease			Myo-inositol	0.801	1.397
Creatine	0.850	1.100	N-Acetylaspartate	0.723	1.038
Phosphocreatine	0.850	1.100	N-Acetylaspartylglutamate	0.723	1.038
Glycerophosphocholine	0.780	1.201	Scyllo-inositol	0.476	1.312
Phosphocholine	0.780	1.201	Taurine	0.882	1.013
γ-Amino Butyric Acid	0.679	1.390	APOE4		
Glutamate	0.887	1.224	Aspartate	1.028	1.168
Glutamine	0.887	1.224	Glycerophosphocholine	0.965	1.019
Myo-inositol	0.810	1.190	Phosphocholine	0.965	1.019
N-Acetylaspartate	0.756	1.240	γ-Amino Butyric Acid	0.513	1.183
N-Acetylaspartylglutamate	0.756	1.240	Glucose	0.971	1.028
Essential Tremor			Glutamate	0.836	1.126
Creatine	0.924	1.053	Glutamine	0.909	1.232
Phosphocreatine	0.924	1.053	Glutathione	0.834	1.103
Glycerophosphocholine	0.851	1.044	Myo-inositol	0.959	1.092
Phosphocholine	0.851	1.044	N-Acetylaspartate	0.895	1.063
γ-Amino Butyric Acid	0.802	1.218	N-Acetylaspartylglutamate	0.895	1.063
Glutamate	1.050	1.434			
Glutamine	1.050	1.434			
N-Acetylaspartate	0.919	1.136			
N-Acetylaspartylglutamate	0.919	1.136			

Supplemental Table 3: Clinical population scaling factors used to generate synthetic spectra. In each case the simulated concentration for a given clinical spectrum was determined by a uniformly sampled concentration drawn from the ranges shown in Supplemental Table 2, multiplied by a scaling factor determined by a uniformly sampled scalar from these ranges provided in Supplemental Table 3.

	Synthetic Range (ms)			Synthetic Range (ms)	
**Metabolite**	**Low**	**High**	**Metabolite**	**Low**	**High**
Acetate	0.00	0.00	Macromolecule 1.67	20.00	60.00
Alanine[*]	100.00	250.00	Macromolecule 2.04	20.00	60.00
Ascorbate	100.00	250.00	Macromolecule 2.26	20.00	60.00
Aspartate	120.15	204.55	Macromolecule 2.56	20.00	60.00
Adenosine Triphosphate	0.00	0.00	Macromolecule 2.70	20.00	60.00
β-Hydroxybutyrate	0.00	0.00	Macromolecule 2.99	20.00	60.00
β-Hydroxyglutarate	0.00	0.00	Macromolecule 3.21	20.00	60.00
Citrate	0.00	0.00	Macromolecule 3.62	20.00	60.00
Creatine 3.03	164.08	242.70	Macromolecule 3.75	20.00	60.00
Creatine 3.91	135.18	213.80	Macromolecule 3.86	20.00	60.00
Creatine 6.65	164.08	242.70	Macromolecule 4.03	20.00	60.00
Cysteine	0.00	0.00	Myo-inositol[†]	139.80	219.58
Ethanolamine	0.00	0.00	N-Acetylaspartate	242.70	320.17
Ethyl Alcohol	0.00	0.00	N-Acetylaspartylglutamate	132.87	216.11
γ-Amino Butyric Acid	77.37	161.77	Phosphocholine	100	250
Glucose	100.00	250.00	Phosphocreatine 3.03	130	210
Glutamine	103.96	184.89	Phosphocreatine 3.93	100	180
Glutamate	140.96	219.58	Phosphocreatine 6.58	130	210
Glycerophosphocholine	198.77	278.54	Phosphocreatine 7.30	130	210
Glutathione	108.59	188.36	Phosphoethanolamine	100	250
Glycine	121.31	204.55	Phosphoethyl Alcohol	0.00	0.00
Glycerol	0.00	0.00	Phenylalanine	0.00	0.00
Histamine	0.00	0.00	Scyllo-inositol	100	250
Histidine	0.00	0.00	Serine	0.00	0.00
Homocarnosine	0.00	0.00	Taurine	151.37	231.14
Lactate	142.12	226.52	Threonine	0.00	0.00
Macromolecule 0.92	20.00	60.00	Tryptophan	0.00	0.00
Macromolecule 1.21	20.00	60.00	Tyrosine	0.00	0.00
Macromolecule 1.39	20.00	60.00	Valine	0.00	0.00

Supplemental Table 4: T_2 Relaxation time ranges in milliseconds for the healthy brain derived from 1.5 T multiple meta-regression preliminary to (Gudmundson et al., 2023). Relaxation times were sampled uniformly between the low and high values.

References:

Gudmundson, A. T., Koo, A., Virovka, A., Amirault, A. L., Soo, M., Cho, J. H., Oeltzschner, G., Edden, R. A. E., & Stark, C. E. L. (2023). Meta-analysis and open-source database for in vivo brain Magnetic Resonance spectroscopy in health and disease. Analytical Biochemistry, 676, 115227. https://doi.org/10.1016/j.ab.2023.115227

Marjańska, M., Deelchand, D. K., Kreis, R., Alger, J. R., Bolan, P. J., Borbath, T., Boumezbeur, F., Fernandes, C. C., Coello, E., Nagraja, B. H., Považan, M., Ratiney, H., Sima, D., Starčuková, J., Soher, B. J., Wilson, M., & van Asten, J. J. A. (2022). Results and interpretation of a fitting challenge for MR spectroscopy set up by the MRS study group of ISMRM. Magnetic Resonance in Medicine, 87(1), 11–32. https://doi.org/10.1002/mrm.28942
